# ssc-cdi: A Memory-Efficient, Multi-GPU Package for Ptychography with Extreme Data

**DOI:** 10.3390/jimaging10110286

**Published:** 2024-11-07

**Authors:** Yuri Rossi Tonin, Alan Zanoni Peixinho, Mauro Luiz Brandao-Junior, Paola Ferraz, Eduardo Xavier Miqueles

**Affiliations:** Brazilian Synchrotron Light Laboratory, Brazilian Center for Research in Energy and Materials (CNPEM), Campinas 13085-970, Brazil; alan.peixinho@lnls.br (A.Z.P.); paola.ferraz@lnls.br (P.F.)

**Keywords:** ptychography, python, imaging, software, X-ray, synchrotron, sirius, HPC, GPU, CDI

## Abstract

We introduce ssc-cdi, an open-source software package from the Sirius Scientific Computing family, designed for memory-efficient, single-node multi-GPU ptychography reconstruction. ssc-cdi offers a range of reconstruction engines in Python version 3.9.2 and C++/CUDA. It aims at developing local expertise and customized solutions to meet the specific needs of beamlines and user community of the Brazilian Synchrotron Light Laboratory (LNLS). We demonstrate ptychographic reconstruction of beamline data and present benchmarks for the package. Results show that ssc-cdi effectively handles extreme datasets typical of modern X-ray facilities without significantly compromising performance, offering a complementary approach to well-established packages of the community and serving as a robust tool for high-resolution imaging applications.

## 1. Introduction

Coherent Diffractive Imaging (CDI) encompasses a family of lensless imaging techniques where the final resolution is no longer limited by the quality of optical components but by the wavelength of radiation and the detector size [1,2]. CDI techniques also go beyond traditional absorption imaging, providing both absorption and phase contrast information. Since the phase signal can be orders of magnitude larger than the absorption signal, phase contrast is a powerful tool for exploring materials with low absorption, such as soft matter samples [3]. Nevertheless, these advances come with a cost in experimental and algorithmic complexity. As the name suggests, CDI requires the use of coherent illumination, which is not always trivial to obtain, and measurements suffer from the phase problem [4] since X-ray detectors only measure the intensity of a signal, therefore loosing phase information.

Ptychography is a variation of CDI that offers a robust solution to the phase problem [5], and has been extremely successful in the X-ray and electron communities [6,7,8]. The increase in robustness comes from the redundancy in measurements obtained through scanning the sample with the coherent beam and making sure that each illuminated region overlaps with neighboring ones. The data redundancy not only makes the phase problem mathematically better posed, but also allows the deconvolution of the sample (referred to as the object) and the incoming wavefront (referred to as the probe) [9]. This approach is particularly effective because it eliminates the requirement for a fully coherent and clean wavefront, as was originally necessary in plane wave CDI (PWCDI) [10]. This relaxation in wavefront requirements eases wavefront engineering, allowing for enhanced beamline flux through focusing, and it places ptychography as a promising method for beam characterization and diagnostics [11]. The need for scanning naturally increases experimental complexity, as the probe positions at the object need to be precisely determined and the beam is assumed to remain stable throughout the experiment. The research community has been successful in circumventing issues that arise when these assumptions are no longer true. Examples are the introduction of position-correction algorithms [12,13,14,15], mixed-states or probe modes [16,17] and non-constant probe during scan [18]. Several ptychography algorithms have been proposed for solving the phase problem, namely the Ptychographic Iterative Engine (PIE) family [19,20,21], Difference Map (DM) [9], Relaxed Averaged Alternating Reflection (RAAR) [22,23] and Maximum Likelihood (ML) [24,25,26,27], among others [12,28,29,30]. Currently, the X-ray community offers many software packages implementing these algorithms, each with a different software architecture and optimization strategy [31,32,33,34,35,36,37,38,39,40].

Ptychography has a relatively recent history at the Brazilian Synchrotron Light Laboratory. Its previous light source, UVX [41], ceased operation in 2019. It was a second-generation machine and therefore did not offer enough coherence for CDI. With Sirius, the new fourth-generation light source of LNLS [42], two beamlines already offer ptychography to users: CARNAÚBA [43] and CATERETÊ [44]. The technique is also of interest for the EMA [45] and MOGNO [46] beamlines as a method for beam characterization. Ptychography will be used in future imaging experiments at LNLS, including in upcoming beamlines for the Orion laboratory [47].

The biggest issue with ptychography arguably lies in its cost. The scanning measurements place a heavy burden on experimental time, data storage and computational processing. This problem becomes even more evident in fourth-generation light sources, as the increased photon flux permits much shorter acquisition times, and the use of large-area detectors [48] generates vast amounts of data. Even with powerful computing nodes equipped with modern general-purpose Graphics Processing Unit (GPU) accelerators, reconstructing a single high-resolution image can take several minutes, and processing an entire tomography dataset may extend to several hours. Moreover, regardless of processing speed, the large volumes of data can pose significant challenges for data processing. Specifically, when a large field-of-view is required in ptychography, the number of measurements for current detector sizes may exceed memory capacity. Consequently, the implementation of High Performance Computing (HPC) strategies is necessary for improving both processing speed and data management.

In this work, we present ssc-cdi, a software package from the Sirius Scientific Computing (ssc) family [49,50,51] that encompasses reconstruction algorithms for ptychography. But why another package? The scientific computing group at LNLS supports beamlines with mathematical modeling and software packages for data processing, particularly those focused on imaging. Since LNLS/SIRIUS serves a relatively new user community for X-ray imaging in South America, a thorough understanding and control over techniques are crucial to consolidate scientific and technical knowledge locally, ensuring a successful beamtime delivery to the user community. Furthermore, the development of local expertise maximizes scientific throughput, enabling the conception of new scientific questions. While it is possible to provide various scientific processing pipelines for specific imaging beamlines by leveraging external tools like PtyPy [31] or PyNX [33], a deep understanding of the computational challenges in conjunction with the beamline allows for the development of customized strategies tailored to the facility’s needs. In this context, the package presented here addresses performance issues, optimizing the use of the computational resources available at LNLS [52]. Furthermore, the fast development of the data-transfer bandwidth between CPU and GPU makes a strong case for exploring CPU memory when dealing with extreme data. Although the amount of Video Random Access Memory (VRAM) in modern GPUs has also greatly increased [53], it is still far from the available Random Access Memory (RAM). Due to the limited availability of HPC nodes for each beamline at LNLS, for instance, we focus on efficiency using a single computing node. We also demonstrate that this can be an advantage for running ptychography outside of an HPC environment.

ssc-cdi is written in Python and offers multiple reconstruction engines for ptychography, namely rPIE, mPIE, Alternating Projections (AP), Relaxed Averaged Alternating Reflection (RAAR) and Maximum Likelihood (ML). All engines are accelerated using single-GPU with cupy [54] and most offer a C++/CUDA back-end with single and multi-GPU functionality. Implementing engines directly in C++/CUDA, despite requiring substantial effort, provides enhanced control over memory management and enables significant advantages in terms of speed and efficiency. The engines also allow for multi-mode probe decomposition [16] and a position-correction approach in CUDA using the Annealing method [13]. In this work, we present an overview both of ptychography and the ssc-cdi package, which has been used for reconstructions at SIRIUS since 2021 [43,44,55]. We detail its HPC strategy and benchmark it against other well-established Python packages, PtyPy and PyNX, to provide an overview of strengths and limitations relative to the state-of-the-art. We show that ssc-cdi consists in a good alternative for processing large datasets without considerably compromising performance, offering a complementary approach to other packages of the community and serving as a robust tool for high-resolution imaging applications.

## 2. Materials and Methods

Ptychography aims at recovering the object O(r) and probe P(r) from a series of *N* intensity measurements $I_{i}$, 1<i<N, at recorded positions $r_{i}=\left(x_{i},y_{i},z\right)$. In what follows, we provide an overview of the main algorithms for solving the phase problem, referred to here as engines.

For X-rays of wavelength λ, the complex refractive index is given by n(r)=1−δ(r)+iβ(r). Within the projection approximation [56], the object is related to real and imaginary refractive indexes, δ(r) and β(r), by the X-Ray Transform R [57]:

(1) $$O\left(r\right)=e^{-kR\{\beta (r)\}}e^{-ikR\{\delta (r)\}},$$

where k=2π/λ and R is performed over the beam direction (*z* axis). For a successful reconstruction, ptychography assumes known scan positions, constant O(r) and P(r) in time, and the multiplicative approximation for the wavefront in the output plane of the sample:

(2) $$\psi_{i}\left(r\right)=P\left(r\right)O\left(r-r_{i}\right).$$

Each scan consists of an intensity measurement, given by the absolute squared value of the propagated wavefront:

(3) $$I_{i}\left(u,v\right)={\left|D_{d}\left\{\psi_{i}\left(r\right)\right\}\right|}^{2},$$

where u,v are the transversal coordinates in the detector plane. The operator $D_{d}$ indicates a free-space propagator by a distance *d*. The form of the propagator depends on the experimental geometry: in the far-field, $D_{d}$ is simply the Fourier transform F; in the near-field, the propagator may assume different forms which depend on the sampling conditions [58,59], the most common being the Angular Spectrum method. Near-field ptychography usually requires less measurements at the cost of a structure-rich probe [60], or the use of multiple scanning planes for successful convergence [61]. Ptychography engines can be broadly categorized into two categories: projection algorithms and cost–function optimization. We describe both in the following sections.

### 2.1. Projection Algorithms

Projection-based algorithms iteratively enforce the measured intensity to be the squared wavefront amplitude at the detector plane. Let *M* be the set of all wavefronts containing the measured magnitudes $\sqrt{I_{i}}$. Since multiple wavefronts $\psi_{i}$ may satisfy the forward model (Equation (3)), projecting the wavefront estimates to M brings them closer to the solution. It can be shown that a projection operation amounts to [62]

(4) $$\Pi_{M}\left\{\psi \right\}=\sqrt{I_{i}}\frac{\psi }{\left|\psi \right|}.$$

Hence, the updated wavefront at the object exit plane is given by

(5) $$\psi_{i}^{\prime }\left(r\right)=D_{d}^{-1}\Pi_{M}D_{d}\left\{\psi_{i}\left(r\right)\right\}.$$

The above wavefront update is the one used in the Alternating Projections (AP) and PIE engines. DM and RAAR propose alternative approaches with different convergence characteristics [9,22]:

(6) $$\psi_{i}^{\prime }=\psi_{i}+\Pi_{M}\left\{2\Pi_{O}\left\{\psi_{i}\right\}-\psi_{i}\right\}-\Pi_{O}\left\{\psi_{i}\right\},$$

(7) $$\psi_{i}^{\prime }=\beta \left(\psi_{i}+\Pi_{M}\left\{2\Pi_{O}\left\{\psi_{i}\right\}-\psi_{i}\right\}\right)+\left(1-2\beta \right)\Pi_{O}\left\{\psi_{i}\right\},$$

respectively, where 0≤β≤1 is a relaxation parameter. $\Pi_{O}$ is the object consistency projector, which amounts to the multiplicative approximation of Equation (2). Note that the DM and RAAR update functions coincide for β=1. The error of each algorithm iteration can be calculated as a normalized mean squared error between the measured intensities and the updated wavefront magnitude at the detector plane:

(8) $$\epsilon =\frac{\sum_{i}(I_{i}-|D_{d}\left\{\psi_{i}^{\prime }\left(r\right)\right\}{{|}^{2})}^{2}}{\sum_{i}I_{i}}$$

After the wavefront update step, one uses $\psi_{i}^{\prime }$ to separate the wavefronts into an object and a probe component. The PIE approach is based on a gradient descent-like step [16] and requires a sequential update of O(r) and P(r) after each wavefront update:

(9) $$O^{\prime }\left(r-r_{i}\right)=O\left(r-r_{i}\right)+w_{i}^{o}\left(r\right)P^{*}\left(r\right)\left(\psi_{i}^{\prime }\left(r\right)-\psi_{i}\left(r\right)\right),$$

(10) $$P^{\prime }\left(r\right)=P\left(r\right)+w_{i}^{p}\left(r\right)O^{*}\left(r-r_{i}\right)\left(\psi_{i}^{\prime }\left(r\right)-\psi_{i}\left(r\right)\right),$$

where * indicates complex conjugate and the weights $w_{i}^{o}$ and $w_{i}^{p}$ are space-dependent factors that differ for each variation of the engine (PIE, ePIE and rPIE) [21]. The update for PIE algorithms is usually performed in random order for the indices *i*, making it stochastic in nature. ssc-cdi implements the mPIE algorithm, which consists of using rPIE update functions

(11) $$O^{\prime }\left(r\right)=O\left(r\right)+s_{o}\frac{P^{*}\left(r\right)\left(\psi^{\prime }\left(r\right)-\psi \left(r\right)\right)}{\left(1-r_{o}\right)|P\left(r\right){|}^{2}+r_{o}{\left|P\left(r\right)\right|}_{\max}^{2}},$$

(12) $$P^{\prime }\left(r\right)=P\left(r\right)+s_{p}\frac{O^{*}\left(r-r_{i}\right)\left(\psi^{\prime }\left(r\right)-\psi \left(r\right)\right)}{\left(1-r_{p}\right)|O\left(r-r_{i}\right){|}^{2}+r_{p}{\left|O\left(r-r_{i}\right)\right|}_{\max}^{2}},$$

followed by optional momentum acceleration, as proposed by [21]. The constants $s_{o}$, $s_{p}$, $r_{o}$ and $r_{p}$ are tunable constants related to the step size and regularization of the update functions, respectively.

The sequential nature of the PIE update function means it is not easily parallelizable. A more efficient approach proposed by [9] is used in the AP, DM and RAAR algorithms, which allows for updating all wavefronts in parallel, prior to the object and probe updates:

(13) $$O^{\prime }\left(r\right)=\frac{\sum_{i}^{N}P^{*}\left(r-r_{i}\right)\psi \left(r\right)}{\sum_{i}^{N}{\left|P\left(r-r_{i}\right)\right|}^{2}},$$

(14) $$P^{\prime }\left(r\right)=\frac{\sum_{i}^{N}O^{*}\left(r+r_{i}\right)\psi \left(r+r_{i}\right)}{\sum_{i}^{N}{\left|O\left(r+r_{i}\right)\right|}^{2}}.$$

### 2.2. Cost–Function Optimization

Solving ptychography directly from the minimization of a function was proposed in the early days of ptychography with a general cost–function that minimizes the difference between observed and expected intensities [12]:

(15) $$C=\sum_{i=1}^{N}\sum_{u,v}M_{i}\left(u,v\right){\left\{{\left|D_{d}\left\{\psi_{i}\left(r\right)\right\}\right|}^{2\gamma }-I_{i}{\left(u,v\right)}^{\gamma }\right\}}^{2},$$

where $M_{i}$ consists of a binary mask to ignore dubious pixels or missing data. When the exponent γ is set to 1/2 or 1, the cost function approximates the maximum likelihood estimators for Poisson and Gaussian counting statistics, apart from additive and multiplicative constants [24,27]:

(16) $$L_{\mathrm{Poisson}}=\sum_{i}^{N}M_{i}\left(u,v\right){\left(\sqrt{{\left|D_{d}\left\{\psi_{i}\left(r\right)\right\}\right|}^{2}}-\sqrt{I_{i}}\right)}^{2},$$

(17) $$L_{\mathrm{Gaussian}}=\sum_{i}^{N}M_{i}\left(u,v\right){\left({\left|D_{d}\left\{\psi_{i}\left(r\right)\right\}\right|}^{2}-I_{i}\right)}^{2}.$$

The incorporation of such noise models contributes to the refinement of reconstructions. Different optimization methods have been proposed for recovery of object and probe in these cases [24,26]. Notwithstanding their success, such methods suffer from a high computational cost compared to projection-based algorithms. In recent years, cost–function minimization has resurged as a promising tool in the community [27,38,39,63] with the advancement in computing power of GPUs and the establishment of libraries such as Theano [64], PyTorch [65] and TensorFlow [66] that allow easy computation of derivatives via Automatic Differentiation and offer an accessible interface to GPUs.

### 2.3. HPC Strategy

Ptychographic reconstruction poses a significant computational challenge due to the iterative nature of the algorithms and especially to the large datasets required for achieving high resolution and reconstructing large samples. The use of parallel strategies, especially through GPU acceleration, is already commonplace for many of the available packages. For instance, PtyPy [31], SHARP [32], PyNX [33] and PtyGer [35] provide GPU accelerated engines. However, many implementations suffer from a significant memory footprint in order to achieve such speed up, and despite the significant increase in available VRAM of commercial GPUs in recent years, processing large datasets remains challenging.

In this context, ssc-cdi primarily focuses on developing fast yet memory-efficient GPU software for ptychography reconstruction, enabling rapid processing without excessive computational overhead. The approach of loading batches from CPU on demand (lazy loading) has the potential to lower the cost of entry-level hardware, making the technique more accessible. Additionally, we prioritize simplicity in code structure and processing pipelines. Our approach involves processing all scanning points of a large field-of-view dataset and synchronizing the data within VRAM, rather than performing parallel ptychography reconstruction of subsets of the whole dataset and subsequently stitching the results, as adopted by PyNX. We avoid the stitching approach for a few reasons. It requires precise knowledge of scan coordinates, removing phase-ramp (see Appendix A), and only then combining the resulting images. From our experience, phase-ramp corrections can be challenging to obtain for samples that span the entire scan field-of-view or that present low contrast. Overcoming these problems may require manually processing data later on, demanding valuable time of users for them to obtain satisfactory results. Even though our approach results in a decrease in processing speed, we demonstrate in the Section 3 that the on demand transfer from RAM to VRAM justifies the trade-off.

To achieve speed with memory efficiency, C++/CUDA is the language of choice for implementing our engines, as it allows for tight control of all memory and low-level access to GPU. Furthermore, using CUDA in association with high-level Python wrappers can significantly mitigate the development cost while maintaining the achieved benefits.

Most of the data involved in the reconstruction process can be efficiently managed within VRAM. For instance, both object *O* and probe *P* are manageable in size even with large-area detectors when using 32-bit float precision for the real and imaginary parts (typically dozens of MBs). However, one significant exception is the list of measurements *I*, which can easily reach hundreds of gigabytes for experiments with high-resolution or large field-of-view. To address this, our engines use smaller, manageable batches of measurements with size *B*, which are transferred to the GPU on demand. We note that performance decreases from this approach can be partly mitigated by employing contiguous page-locked memory (pinned memory).

The projection $\Pi_{M}$ is a critical component for the implementation of all algorithms within the package. To compute the Fourier transforms associated with it, we rely on NVIDIA’s CUFFT [67], an optimized CUDA library that implements the Fast Fourier Transform (FFT), for several use cases in GPU-accelerated systems. It provides a ready to use, fast and relatively memory-efficient implementation, while consuming roughly 50% of spent GPU kernel time. For the majority of our custom CUDA kernels, straightforward implementations were sufficient to guarantee good performance, with no apparent bottlenecks observed. Two exceptions required a more elaborate approach, nonetheless.

The first exception is computing the error ϵ (Equation (8)), which involves aggregating the element-wise difference between measured diffraction patterns and computed diffraction patterns. This requires atomic summation of the squared residuals, which we address with a parallel reduction strategy in shared memory, with a block size of 64 to optimize performance and ensure efficient computation.

Second, for those algorithms that implement the update step—Equations (13) and (14)—after computation over all diffraction patterns (AP, DM and RAAR), the projector $\Pi_{M}$ can be concurrently applied to each wavefront $\psi_{i}$, and the updated $\psi_{i}^{\prime }$ batch can then be used for computing the new object and probe matrices. We implement yet another acceleration step of this update [55] by separately accumulating the numerator and the denominator in Equations (13) and (14), as it also improves performance.

#### Multi-GPU Implementation

**Figure 1:**
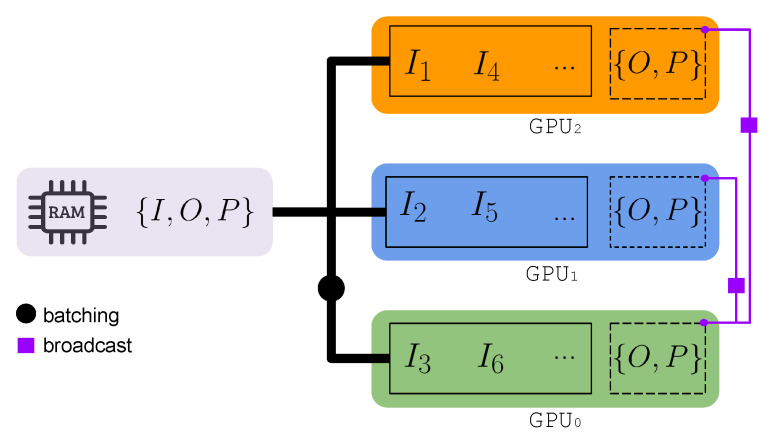
Diagram illustrating the batch distributions of measurements for three GPUs. Each colored block represents data inside of the respective GPU. A batch of B≤N measurements is distributed to the GPU memory, so that the wavefronts are updated in parallel. Once a GPU finishes processing and is made available, a remaining batch of unprocessed data is loaded from RAM. After all batches have been loaded and all the wavefronts updated, the new object and probe matrices are calculated by GPU_0_ and then broadcasted to the other GPUs, so that each of them has faster access to *O* and *P* in the subsequent iteration.

In the multi-GPU scenario, measurements are partitioned into distinct batches, which are then distributed across all available GPUs within a single node (Figure 1). Each GPU processes a unique batch independently. If the number of batches for a given batch size is bigger than the number of available GPUs, the remaining batches await in RAM and are only transferred to VRAM when a GPU becomes available. This approach simplifies the efficient parallelization of AP and RAAR by allowing each GPU to handle a subset of the data.

Synchronization is primarily required during the probe and object update step. In this case, the batch summation of numerator and denominator are computed on each GPU and later aggregated on the first GPU using a binary reduction tree method. This approach combines partial sums recursively—with a logarithmic complexity—over the number of GPUs. After aggregation and subsequent application of Equations (13) and (14), the updated probe and object are broadcast to all GPUs to ensure that each has access to the new matrices, maintaining consistency for subsequent iterations.

The adopted strategy also means that our current implementation of stochastic projections (rPIE and mPIE) is restricted to a single GPU with one measurement per batch, due to the intrinsically sequential nature of these algorithms.

## 3. Results

**Figure 2:**
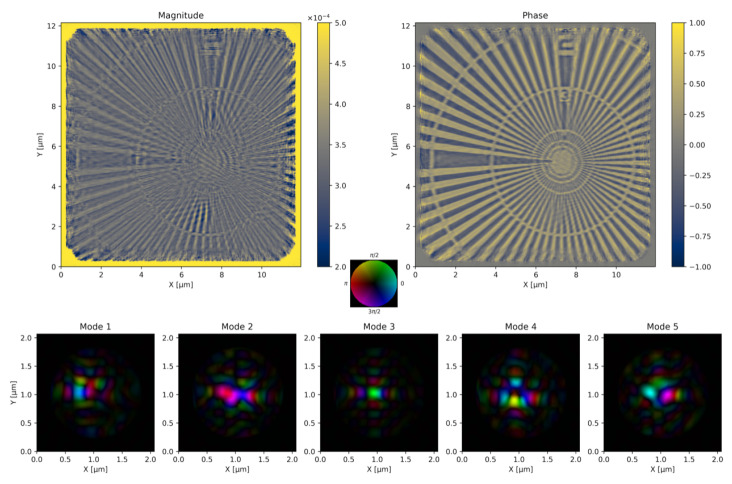
Ptychography reconstruction of a Siemens Star measured at CARNAÚBA beamline. The finest features of the innermost circles are spaced 15nm from each other. The complex probe is shown in an hsv colormap, saturation encoding magnitude and hue encoding the phase.

We first validate ssc-cdi by presenting the reconstruction of a real dataset acquired at CARNAÚBA beamline [68]. A Siemens Star (manufacturer Applied Nanotools Inc., Edmonton, AB, Canada) was measured at 12keV with detector placed at 1.1m from the sample. The diffraction patterns were acquired in fly-scan mode, with a total of 10,100 measurements. The detector pixel size is $55\times 55\;\mu m^{2}$ and an area of 256×256 pixels was used for reconstruction, resulting in an effective pixel of 8nm. Figure 2 shows the reconstructed magnitude and phase of the sample, as well as the probe. The fly-scan acquisition required using at least five probe modes for a satisfactory reconstruction due to the integrating nature of the intensity measurements, which can be equivalently interpreted as an incoherence effect [69]. The object was initialized with random amplitude and constant phase, whereas the first probe mode was set to the inverse Fourier Transform of the averaged diffraction data. We used 100 iterations of rPIE followed by 300 iterations of AP with a loose circular probe support of 1.5μm diameter.

### Benchmarks

To evaluate the performance and effectiveness of ssc-cdi, we conducted a series of benchmarks against well-established packages in the community, namely PtyPy 0.8.0 [31] and PyNX 2023.1.1 [33]. These benchmarks provide a comprehensive understanding of the strengths and limitations of our approach relative to current state-of-the-art methods. All benchmarks were conducted on the LNLS cluster [52], utilizing a NVIDIA DGX A100 system [70] with an AMD EPYC 7742 64-Core Processor, 1 TB of RAM and eight A100 GPUs, each featuring 40 GB of VRAM connected with NVLINK 300 GB/s high-bandwidth and PCIe (Gen4) with 32 GB/s bandwidth. The system employs the NVIDIA driver version 460.27.04, with CUDA 11.2 in an Ubuntu 20.04 operating system. We detail the parameters used in the algorithm calls for each package in the Supplementary File S1.

**Figure 3:**
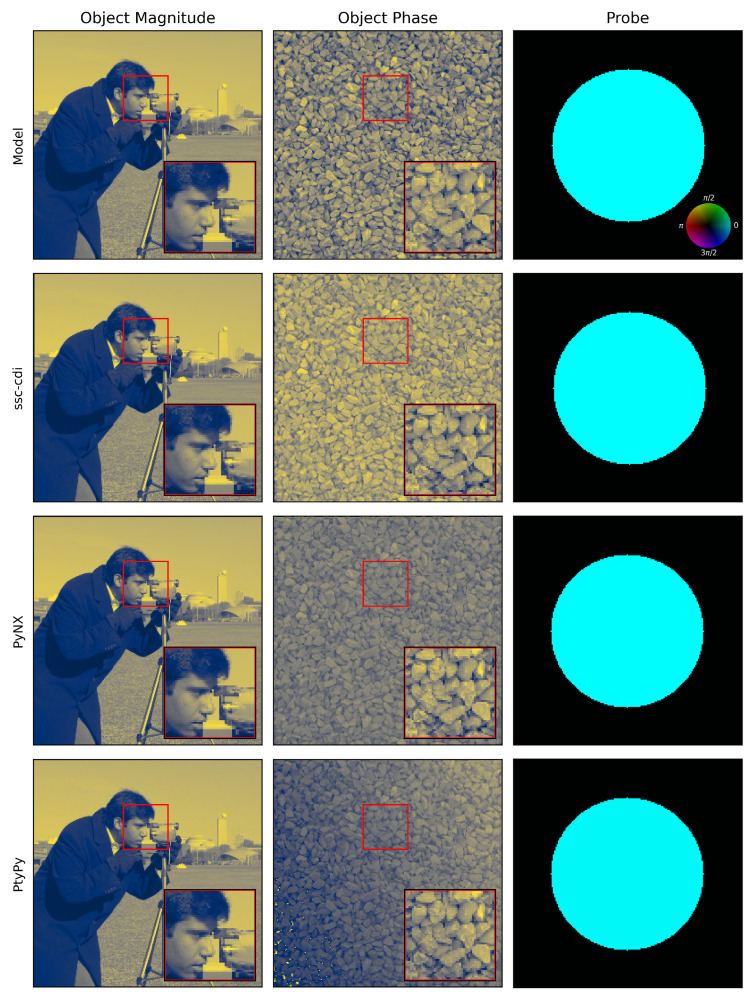
Comparison of the simulated sample against the reconstruction using the DM algorithm from different packages. The insets show a zoomed region from the red square in the object and phase reconstructions. The reconstruction for ssc-cdi used the RAAR algorithm with parameter β=1, such that the update function equals that of DM. For PyNX and PtyPy; we used the DM engine directly. In all cases, the same initial guesses were used: random magnitude and constant phase for the object array, and an inverse Fourier transform of the averaged measurements for the probe.

The initial goal was to ensure that all packages could successfully reconstruct data from a numerical experiment. Subsequently, we compared the speed and scalability of various ptychographic engines across the different packages using the same dataset and reconstruction parameters. The selected dataset for object amplitude and phase was the Camera Man and Gravel test images from [71], with a circular probe of constant phase, as shown in the top row of Figure 3. The object consisted of 400×400 pixels and probe of 180×180 pixels, with a raster grid scan recorded at 400 positions, with normally distributed offsets added to the grid points to avoid the raster grid pathology [9]. For evaluating convergence, we ran 200 iterations of the DM algorithm with each package using the same initial guesses. DM was the algorithm of choice as it is available across all packages, providing a consistent basis for comparison. Figure 3 shows the reconstructed object and probe by the different packages. Convergence was successful in all cases. A loose support for the probe was used, causing the reconstructions to be shifted by different amounts in each case. The position of the probe magnitude center of mass was used to correct for this shift. One can notice a slight difference in the phase ramp for the phase reconstruction in each case, which could arguably be avoided by a more meticulous tuning of engine parameters and initial guesses, or combining different algorithms for refinement.

For the speed and scalability benchmarks, we used the same dataset. In this case, the object and probe matrices were binned or interpolated as necessary to reach the desired matrix dimensions. The reported times do not include the time required for loading data from the disk.

**Figure 4:**
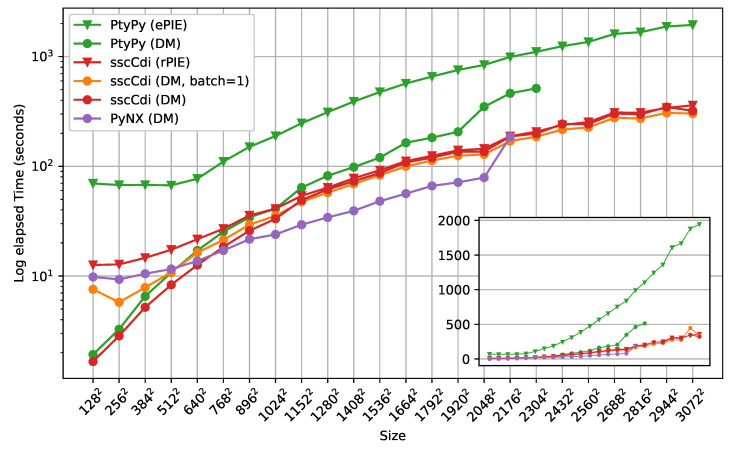
Single GPU performance of DM and PIE algorithms across different packages. The inset shows the same data without log scale on the vertical axis. Missing points on some curves indicate dimensions that were not supported by a specific engine. Note that PyNX does not provide an engine for an algorithm of the PIE family for comparison.

Figure 4 shows the benchmark results as function of measurement size *N*, where the used dataset has dimensions (400,N,N). We compared both the DM and PIE family algorithms. All engines ran 200 iterations from N=128 to N=3072 (the largest area detector dimension at LNLS [48]) in steps of N=128. The missing data points for some of the curves indicate that the engine could not cope with the desired dimensions due to lack of memory. All packages present a variable to control batch size *B* with similar purpose, which we set to B=128, apart from ssc-cdi’s rPIE engine, which is hard-coded for B=1. In terms of speed, ssc-cdi lies in between the performance of PyNX and PtyPy. For the PIE algorithms, both engines ran successfully for all data sizes. This is expected as the sequential nature of PIE implies a small memory footprint and only a single wavefront array needs be stored in memory at a time. For DM, our approach performs faster only for small dimensions, lying in between PtyPy and PyNX for the dimensions above $768^{2}$. We note that the performance of our DM engine eventually converges to the performance of rPIE as the cost associated with data transfer greatly increases with the data size, causing data transfer and Fourier transforms—common operations for both engines—to predominantly influence the overall running time. We show an extra curve for ssc-cdi using DM with batch B=1, which gets closer to the rPIE curve because a larger number of transfers happens in this case. The clear strength of ssc-cdi comes from the batching strategy from RAM to VRAM, which is made evident here. Among the DM engines, ssc-cdi’s was the only one capable of reconstructing large datasets, despite the algorithm’s substantial memory footprint.

**Figure 5:**
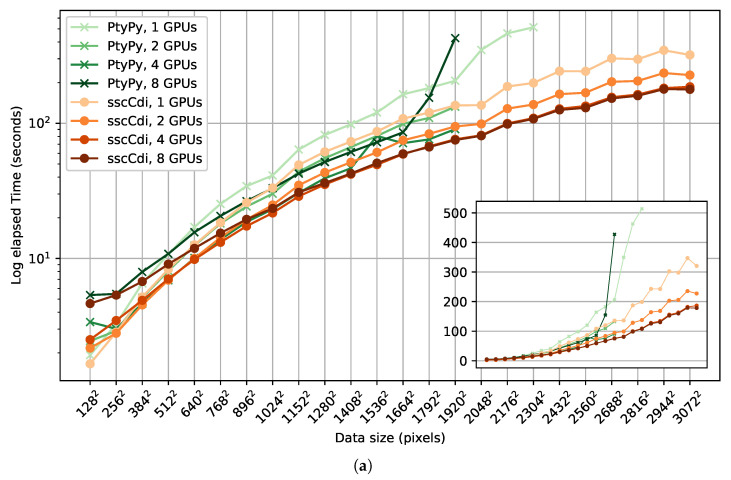

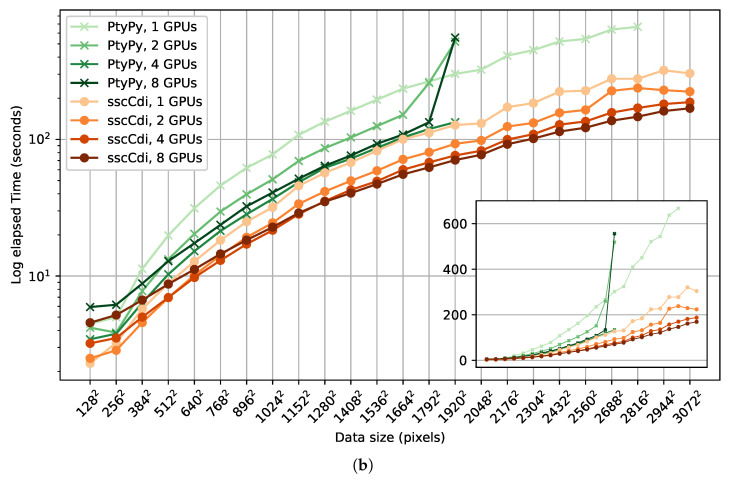
Multi-GPU performance of DM algorithm for ssc-cdi and PtyPy using batch sizes of (**a**) 128 and (**b**) 16. Dimensions that were not supported by an engine are the reason for missing points for some of the curves. The inset plots the same data without log scale on the vertical axis.

We also analyzed multi-GPU performance of the DM engines. A few differences must be noted here. ssc-cdi provides multi-GPU support directly through CUDA, whereas PtyPy offers multi-GPU capability via OpenMPI. We excluded PyNX from the comparison because its multi-GPU approach utilizes the previously mentioned stitching strategy, which is not equivalent to PtyPy and ssc-cdi. We ran 200 iterations of DM using the same data dimensions and input parameters, repeating it for 2, 4 and 8 GPUs. Figure 5a shows results for a batch size B=128. In terms of speed, ssc-cdi is able to reconstruct faster than its PtyPy counterpart for almost all data points, especially for large *N*. In addition, regarding memory management and scalability, ssc-cdi was able to handle all tested data sizes, due to the strategy of lazy loading batches into GPU, which is essential for processing the data volumes currently generated by LNLS beamlines and is further supported by the increasing bandwidth of NVIDIA’s latest architectures.

Since the batch size *B* is crucial for determining how the engine manages large data, we ran a second benchmark for B=16 (Figure 5b). We can see that the scalability of PtyPy indeed improves at the cost of higher execution time. ssc-cdi exhibits equivalent performance when compared to the larger batch case.

Additionally, we observe that the performance gains marginally decrease as more GPUs are utilized, with 4 GPUs providing reasonable compromise. For B=128, using 8 GPUs presents no benefit as expected, since the 400 scan points are divided in only four batches of sizes 128, 128, 128 and 16. Nonetheless, the benefit becomes visible again for B=16, where the scans points are divided into 400/16=25 batches. We note that the point-to-point performance fluctuations in some cases may be partly attributed to the the machines not being used exclusively for our benchmarks during the tests, as machine RAM could be shared with other cluster users.

**Figure 6:**
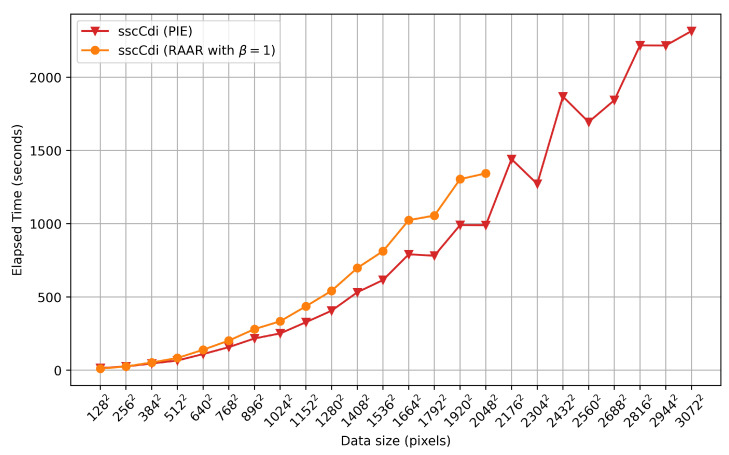
Single GPU performance of ssc-cdi for DM and PIE engines at a conventional machine. RAAR was run with batch size B=1 and managed to run up to a data size of $2048^{2}$.

As a final experiment, we evaluated the performance of the rPIE and DM algorithms across all previously discussed data sizes on a simpler setup, outside the HPC environment. This machine was equipped with an Intel Xeon E5-2630 v3 CPU, 64 GB of RAM, and a single NVIDIA Quadro M4000 GPU with 8 GB of VRAM. The system employs the NVIDIA driver version 460.27.04, with CUDA 12.4 in an Ubuntu 22.04 operating system. Execution times shown in Figure 6 demonstrate that, despite the modest machine specifications compared to HPC environments, ssc-cdi was still able to run rPIE for all data sizes and RAAR up to N=2048 in less than an hour. This finding reinforces the well-balanced trade-offs of our approach, demonstrating the potential use of advanced ptychography algorithms on more accessible hardware and advocating its relevance to the community.

## 4. Discussion

The development of ssc-cdi has been an ongoing effort since Sirius began operations in 2019, with contributions from physicists, mathematicians, and computer scientists. From the outset, the ability to reconstruct large datasets generated at Sirius has been a primary objective guiding its development and we relied on the principle that being able to handle a dataset, albeit at a slower pace, was preferable to being unable to process it at all. The HPC strategy and benchmarks presented in this work demonstrate that ssc-cdi provides a memory-efficient, multi-GPU approach to ptychographic reconstruction, effectively handling measurements from detectors with dimensions up to 3072×3072 pixels without significantly compromising performance. The on demand memory-loading approach is also favored by the fast improvement on CPU–GPU bandwidth currently happening, and it favors the use of ssc-cdi even outside of HPC environments, such as more modest single-node laboratory machines with limited VRAM.

ssc-cdi is publicly available at https://doi.org/10.5281/zenodo.13693178 (accessed on 27 October 2024). It is important to acknowledge that the current release still has limitations compared to some of the established packages within the X-ray imaging community. For example, the package currently does not include a CUDA-based maximum likelihood engine, fast algorithms for position correction [14,15], orthogonal probe relaxation [18], multi-slice approaches [72,73,74] or modern algorithms such as ADMM [29,75] and WASP [30]. These are functionalities left for future releases of ssc-cdi.

Finally, it should be noted that the development of C++/CUDA software was essential to achieving the demonstrated performance. Although the time invested in writing C++/CUDA software is substantial, the effort is justified. Our results show that ssc-cdi  enables memory-efficient reconstructions, achieving performance that is comparable to, or even exceeds, that of well-established tools in the community. Moreover, the development of our own approach has allowed us to create custom solutions tailored to the specific needs of the beamlines at LNLS, with the flexibility to adapt as these beamlines have transitioned from a commissioning phase to operation over the past few years. As implied by its name, ssc-cdi is designed to be more than just a ptychography toolbox. Future releases will include the implementation of multi-GPU distribution for three-dimensional PWCDI reconstructions [76], which will be crucial for CDI reconstruction of large volumes, as well as the addition of new engines and improvements for ptychography.

## Data Availability

The ssc-cdi package is publicly available in Zenodo (https://doi.org/10.5281/zenodo.13693178, accessed on 27 October 2024) or upon request with one of the corresponding authors.

## Supplementary Materials

The following supporting information can be downloaded at: https://www.mdpi.com/article/10.3390/jimaging10110286/s1, Supplementary File S1: Benchmark details.

## Abbreviations

CDI	Coherent Diffractive Imaging
PWCDI	Plane-wave Coherent Diffractive Imaging
PIE	Ptychographic Iterative Engine
AP	Alternating Projections
DM	Difference Map
RAAR	Relaxed Averaged Alternating Reflection
ML	Maximum Likelihood
GPU	Graphics Processing Unit
HPC	High Performance Computing
SSC	Sirius Scientific Computing
RAM	Random Access Memory
VRAM	Video Random Access Memory
FFT	Fast Fourier Transform
BP	Backprojection
FBP	Filtered Backprojection

## Appendix A

In ptycho-tomography experiments, one rotates the sample around the vertical axes, performing a ptychographic scan in between each rotation. After all 2D images (referred to here as sinogram projections) are obtained, one may use them in combination with tomography algorithms for reconstructing a 3D volume of the sample. Nonetheless, a careful treatment of data is often required prior to using a tomography algorithm [77], especially when working with phase-contrast images. ssc-cdi offers useful Python functions for users to process data prior to tomography.

First, the extracted phase signal is periodic with period 2π. For strongly scattering samples, this can easily cause phase wrapping of images. ssc-cdi optimizes the phase-unwrapping algorithm [78] by parallelizing the process across multiple CPU cores for each projection, significantly reducing the unwrapping procedure by several times.

One important aspect of ptychography is the ambiguities in its solution. Given the multiplicative approximation, we have the equivalence

(A1) $$\psi \left(r\right)=P\left(r\right)O\left(r-r_{i}\right)=\left[Ae^{iR\cdot r}P\left(r+t\right)\right]\left[A^{-1}e^{-iR\cdot r}O\left(r-r_{i}+t\right)\right],$$

that is, the retrieved object and probe may present an amplitude offset *A* and a first-order phase-ramp R(x). Additionally, the shift invariant property of the Fourier transform added to the fact that only the amplitude of the signal is measured makes the reconstruction invariant to a translation t in real space. Consequently, one needs to equalize the projections by removing phase-ramps from each projection [77] prior to tomography. ssc-cdi further enhances this method through an implementation across multiple CPU cores. We observe that successful removal of phase-ramps usually requires a linear fit of the background both to the left and right of the sample. For that, a binary mask is used to select multiple regions of air around the sample. Samples with a single interface to air in the measured field-of-view usually prove difficult to equalize.

A potential workaround to significantly reduce processing time is to skip the equalization process and perform tomography directly from the wrapped phase [77]. This method requires differentiation of the phase signal, which may be problematic for low-contrast samples with bad signal-to-noise ratio since noise is amplified by the differentiation. ssc-cdi also provides a function call for preparing the phase-wrapped sinogram using the Hilbert transform. Filtered Backprojection (FBP) allows the recovery of the 3D refractive index δ(z,y,x) from a set of phase projections ϕ [79]:

(A2) $$-\frac{2\pi }{\lambda }\delta \left(z,y,x\right)=\int_{0}^{\pi }F^{-1}\left\{|u|F\left\{\phi \right\}\right\}\left(\theta ,y,x\cos\theta +z\sin\theta ,y\right)d\theta ,\;\;\;\forall y.$$

Letting H be the Hilbert transform and sgn the signal function, we can use the following properties

(A3) $$F\left\{\frac{\partial \phi }{\partial x}\right\}=i2\pi uF\left\{\phi \right\},$$

(A4) F{H(u)}=−isgn(w)F{u},

to further simplify Equation (A2) and show that δ(z,y,x) is proportional to the backprojection (BP) of the Hilbert transform of the phase gradient:

(A5) $$\delta \left(z,y,x\right)=-\frac{\lambda }{4\pi^{2}}\int_{0}^{\pi }H\left\{\frac{\partial \phi }{\partial x}\left(\theta ,y,x\cos\theta +z\sin\theta \right)\right\}d\theta ,\;\;\;\;\forall y.$$

At last, the translation ambiguities, together with instabilities of the mechanical stages during rotation of the sample, make it necessary for alignment of sinogram projections. We do not discuss such a process here, as it has been thoroughly presented elsewhere [77,80]. In our pipelines, alignment functions are offered as part of the ssc-raftpackage, publicly available in [49].
