# Supplementary material for: ssc-cdi: A Memory-Efficient, Multi-GPU Package for Ptychography with Extreme Data
Source: J Imaging. 2024 Nov 7;10(11):286. doi: 10.3390/jimaging10110286 (PMC11595696; doi:10.3390/jimaging10110286)
Supplement: Supplementary file 1 [file jimaging-10-00286-s001.zip › Supplementary File S1.pdf]

Supplementary File S1: Benchmark details

Y. R. Tonin, A. Z. Peixinho, M. L. Brandao-Junior, P. Ferraz and E. X. Miqueles

Abstract

In this supplement, we provide some details about the benchmarks, so that results may be reproduced by the community if desired.

1 Timing in each package

We detail ahead the main function calls and where time was tracked. For all benchmarks, data was first loaded to RAM before calling the engines. That includes the diffraction patterns, positions, initial object and initial probe arrays.

For `ssc-cdi`, time was tracked before and after calling the `sscCdi.call_ptychography()` function, whereas for `PyNX`, we tracked the calls of `p=Ptycho()` and `DM()**niter*p`.

`PtyPy`, on the other hand, saves the time of different portions of the code by setting the `io.benchmark="all"` flag. These portions are named: "data_load", "engine_init", "engine_prepare", "engine_iterate" and "engine_finalize". For all `PtyPy` benchmarks presented, we excluded "data_load" time and summed the others.

2 Listing of input parameters

Ahead we present how the input parameters were set in each benchmark. As mentioned in the text, all packages present a variable to control batch size B with similar purpose. In the listings ahead, B is passed in the `frames_per_block` variable.

2.1 ssc-cdi

```
input_dict = {
"hdf5_output": "./output.h5",
"CPUs": 32,
"GPUs": [0],
"fresnel_regime": False,
"energy": 10, # energy in keV
"detector_distance": 15,  # meters
"distance_sample_focus": 0, # float.
"detector_pixel_size": 55e-6, # meters
"binning": 1, # if 1, no binning occurs.
"position_rotation": 0, # angle in radians.
"object_padding": 20, # pixels.
"incoherent_modes": 1, # int.
"probe_support": {"type": "circular",  "radius": 64,  "center_y": 0,
    "center_x": 0}
}
```

```
# set engine parameters for ssc-cdi (PIE)
input_dict["algorithms"] =  {"1": {"name":"PIE",
                              "iterations": niter,
                              "step_object": 1.0,
                              "step_probe": 1.0,
                              "batch": frames_per_block, # not used for
                                 PIE. batch=1 by standard
                              "momentum_obj":0.0,
                              "momentum_probe":0.0,
                              "regularization_object": 1.0,
                              "regularization_probe": 1.0,
                              }}

# Run ssc-cdi
tic = time.time()
recon_obj, recon_probe, recon_positions, output_dict, recon_error =
    sscCdi.call_ptychography(input_dict, data, positions,
    initial_obj=initial_obj.copy(), initial_probe=initial_probe.copy() )
toc = time.time() - tic
print("Elapsed time for PIE(sscCdi): {} sec".format(toc))
```

On the other hand, for DM algorithm we simply change the "algorithms" key to RAAR with $\beta = 1$:

```
input_dict["algorithms"] = {"1": {"name":"RAAR",
                          "iterations": niter,
                          "beta": 1.0,
                          "step_object": 0.9,
                          "step_probe": 0.9,
                          "regularization_object": 0.01,
                          "regularization_probe": 0.01,
                          "momentum_obj": 0.0,
                          "momentum_probe": 0.0,
                          "batch": frames_per_block,
                          "position_correction":0,
                          }}
```

2.2 PyNx

```
import pynx.ptycho.cu_operator as cuop
cuop.default_processing_unit.set_stack_size(frames_per_block)

# ... other definitions

# input parameters
wavelength =  2.07e-10
detector_distance = 15
pixel_size_detector =  55e-6
n = data.shape[1]
pixel_size_object = wavelength * detector_distance / pixel_size_detector /
    n
```

```
# create pynx data object
data_pynx = PtychoData(data,
                       positions=(positions_pynx[:,1]*pixel_size_object,
                           positions_pynx[:,0]*pixel_size_object),
                       detector_distance=detector_distance,
                       mask=None,
                       pixel_size_detector=pixel_size_detector,
                       wavelength=wavelength)

tic = time.time()
p = Ptycho(probe=initial_probe, obj=initial_obj, data=data_pynx,
    background=None, nb_frame_total=None)

p = DM(update_object=True,
       update_probe=True,
       zero_phase_ramp=False)**niter * p

toc = time.time() - tic
print('Elapsed time for DM(PyNX): {} sec'.format(toc))
```

2.3 PtyPy

```
ptypy.load_gpu_engines("cupy") # or "cuda"

# Create parameter tree
parameter_tree = u.Param()
parameter_tree.verbose_level = "interactive"
parameter_tree.run = run_ID
parameter_tree.io = u.Param()
parameter_tree.io.home = io_home_path
parameter_tree.io.rfile =  ptycho_final_path
parameter_tree.io.interaction = u.Param(active=False)
parameter_tree.io.autosave = u.Param()
parameter_tree.io.autosave.active = False
parameter_tree.scans = u.Param()
parameter_tree.scans.CateretePtyPyScan = u.Param()
parameter_tree.scans.CateretePtyPyScan.data = u.Param()
parameter_tree.scans.CateretePtyPyScan.name = "BlockFull"
parameter_tree.frames_per_block = frames_per_block
parameter_tree.scans.CateretePtyPyScan.data.name = 'PtydScan'
parameter_tree.scans.CateretePtyPyScan.data.source = 'file'
parameter_tree.scans.CateretePtyPyScan.data.dfile = ptyd_output_path

# benchmarking parameters
parameter_tree.scans.CateretePtyPyScan.data.save = None
parameter_tree.io.benchmark = "all" # Although we are saving all benchmark
    keys, later, we do not account for "data_load" when registering in our
    logs

parameter_tree.engines = u.Param()
parameter_tree.scans.CateretePtyPyScan.sample = u.Param()
parameter_tree.scans.CateretePtyPyScan.sample.model = initial_obj
parameter_tree.scans.CateretePtyPyScan.illumination = u.Param()
```

```
parameter_tree.scans.CateretePtyPyScan.illumination.model = initial_probe
parameter_tree.engines.engine00 = u.Param()
parameter_tree.engines.engine00.name = "EPIE_cupy" # or EPIE_pycuda
parameter_tree.engines.engine00.numiter = niter
parameter_tree.engines.engine00.numiter_contiguous = niter
parameter_tree.engines.engine00.probe_support = None #
probe_update_cuda_atomics = False # False by default
object_update_cuda_atomics = False # True by default
fft_lib = "cuda" # cupy by default

t1 = time.time()
P = ptypy.core.Ptycho(parameter_tree, level=5)
elapsed = time.time() - t1
print(f'Time elapsed: {elapsed/60} min')
```

For DM, the engine parameters were changed to:

```
parameter_tree.engines.engine00 = u.Param()
parameter_tree.engines.engine00.name = "DM_cupy" # other option is
    "DM_cupy"
parameter_tree.engines.engine00.numiter = niter
parameter_tree.engines.engine00.numiter_contiguous = niter
parameter_tree.engines.engine00.overlap_max_iterations = 1
parameter_tree.engines.engine00.alpha = 1.0
parameter_tree.engines.engine00.fourier_power_bound = 0.25
parameter_tree.engines.engine00.probe_support = None
probe_update_cuda_atomics = False # False by default
object_update_cuda_atomics = False # True by default
```
